# *Streptomyces globosus* UAE1, a Potential Effective Biocontrol Agent for Black Scorch Disease in Date Palm Plantations

**DOI:** 10.3389/fmicb.2017.01455

**Published:** 2017-07-31

**Authors:** Esam E. Saeed, Arjun Sham, Zeinab Salmin, Yasmeen Abdelmowla, Rabah Iratni, Khaled El-Tarabily, Synan AbuQamar

**Affiliations:** Department of Biology, United Arab Emirates University Al Ain, United Arab Emirates

**Keywords:** actinomycetes, antibiosis, biocontrol, black scorch, date palm, *Thielaviopsis punctulata*, UAE

## Abstract

Many fungal diseases affect date palm causing considerable losses in date production worldwide. We found that the fungicide Cidely^®^ Top inhibited the mycelial growth of the soil-borne pathogenic fungus *Thielaviopsis punctulata*, the causal agent of black scorch disease of date palm, both *in vitro* and *in vivo*. Because the use of biocontrol agents (BCAs) can minimize the impact of pathogen control on economic and environmental concerns related to chemical control, we aimed at testing local actinomycete strains isolated from the rhizosphere soil of healthy date palm cultivated in the United Arab Emirates (UAE) against *T. punctulata*. The selected isolate can thus be used as a potential agent for integrated disease management programs. In general, the BCA showed antagonism *in vitro* and in greenhouse experiments against this pathogen. The most promising actinomycete isolate screened showed the highest efficacy against the black scorch disease when applied before or at the same time of inoculation with *T. punctulata*, compared with BCA or fungicide application after inoculation. The nucleotide sequence and phylogenetic analyses using the 16*S* ribosomal RNA gene with other *Streptomyces* spp. in addition to morphological and cultural characteristics revealed that the isolated UAE strain belongs to *Streptomyces globosus* UAE1. The antagonistic activity of *S. globosus* against *T. punctulata*, was associated with the production by this strain of diffusible antifungal metabolites i.e., metabolites that can inhibit mycelial growth of the pathogen. This was evident in the responses of the vegetative growth of pure cultures of the pathogen when exposed to the culture filtrates of the BCA. Altogether, the pathogenicity tests, disease severity indices and mode of action tests confirmed that the BCA was not only capable of suppressing black scorch disease symptoms, but also could prevent the spread of the pathogen, as a potential practical method to improve disease management in the palm plantations. This is the first report of an actinomycete, naturally occurring in the UAE with the potential for use as a BCA in the management of the black scorch disease of date palms in the region.

## Introduction

Date palm (*Phoenix dactylifera* L.) is cultivated for its edible fruit and for its value as a shelter to humans, animals, and plants. In the United Arab Emirates (UAE) and in many other countries in the region, this plant also has social, traditional and heritage values (Mahmoudi et al., 2008). Date palm trees are exposed to various pathogenic infections, mainly fungi and phytoplasma, causing serious deleterious diseases as well as significant economic losses (Carpenter and Elmer, 1978). Black scorch disease, also locally known as Medjnoon or Fool, is caused by the fungus *Thielaviopsis paradoxa* (De Seyeres) Hohn or *Thielaviopsis punctulata* (Hennebert) Paulin, Harrington and McNew (de Beer et al., 2014). These soil-borne wound pathogens affect date palm tissues at all ages of growth, over a wide range of date growing areas in the world, causing losses of >50% in newly plantations and fruits (Gariani et al., 1994; Abdelmonem and Rasmy, 2007; Saeed et al., 2016). Previous reports have associated *T. paradoxa* with black scorch disease on date palm in Iraq (Abbas et al., 1997), Saudi Arabia (Al-sharidy and Molan, 2008), Kuwait (Mubarak et al., 1994), Italy (Polizzi et al., 2006) and the United States (Garofalo and McMillan, 2004). Recent studies have identified *T. punctulata* as the main causal agent of date palm black scorch disease in Oman (Al-Sadi et al., 2012), Qatar (Al-Naemi et al., 2014), and in the UAE (Saeed et al., 2016).

Depending on the time of infection and the stage of the disease development, typical symptoms of black scorch are hard black lesions on leaves, inflorescence blight, and trunk and bud rot (Suleman et al., 2001; Zaid et al., 2002; Abbas and Abdulla, 2003; Al-Raisi et al., 2011). Severe symptoms are well-characterized by “neck bending” of the regions affected by the fungal invasions in the terminal bud and heart, leading to the death of the tree. Poor horticultural practices and environmental stresses can also exacerbate the disease development. Studies on *T. paradoxa*- and *T. punctulata*-colonized palm tissues under salinity and drought stresses showed increased severity of black scorch, which eventually resulted in plant death (Suleman et al., 2001). Traditional field management practices such as avoidance of wounds in tissues or tree parts, cutting and burning affected palms have been reported to limit disease incidence (Chase and Broschat, 1993). Appropriate irrigation and fertilization programs are also recommended to minimize disease severity (Zaid et al., 2002).

Chemical application continues to be the major strategy to mitigate the menace of most crop diseases, despite its negative impact on the environment and human health. For example, the fungicide benomyl is highly effective against early infections by date palm pathogens (Zaid et al., 2002). Recently, it has been reported that Score^®^ (difenoconazole) is an effective chemical treatment for black scorch on date palm (Saeed et al., 2016). On the other hand, integrated disease management (IDM) aims at reducing chemical inputs by delivering lower amounts and/or less frequent treatments with chemical fungicides, applied only when necessary (Lopez-Escudero and Mercado-Blanco, 2011). Biological control, also known as biocontrol, can limit the increases in pathogen populations, and often suppress the plant tissue destroying activities of pathogens by other organism(s) (AbuQamar et al., 2017). *In vitro* and *in vivo* experiments using species of *Trichoderma*, *Chaetomium* or their antagonistic products were applied to control black scorch pathogens (Soytong et al., 2005; Sánchez et al., 2007; Ammar, 2011; Al-Naemi et al., 2016).

Actinomycetes are a diverse group of Gram-positive bacteria that exhibit wide morphological differences that range from relatively simple rods and cocci to complex mycelial organization (Locci and Sharples, 1984). Reproduction is by fragmentation of the hyphae or by production of spores. Actinomycetes are ubiquitous, found in habitats such as soils, composts, freshwater, seawater, and cold- and warm-blooded animals. *Streptomyces* spp., a common genus in the order Actinomycetales, are a biologically active component of the soil microflora (Williams and Wellington, 1982). They are mostly found in relatively dry, humic, calcareous soils. *Streptomyces* spp. have been investigated predominantly, because of their dominance, ease of isolation and antibiotics production (Goodfellow and Williams, 1983).

Actinomycetes have also been identified as active biocontrol agents (BCAs) effective against many pathogenic fungi and oomycetes (Lee et al., 2005; El-Tarabily et al., 2009). They are known for their bioactive metabolites, including antibiotics, plant growth factors, vitamins, alkaloids, enzymes and enzyme inhibitors active against their natural enemies (Doumbou et al., 2001; Bressan, 2003; Shahidi et al., 2004; El-Tarabily and Sivasithamparam, 2006; Verma et al., 2011; Baltz, 2016). In general, biological control is an environmentally sustainable strategy that can be employed to manage plant pathogens and improves crop productivity.

In addition, rhizosphere microorganisms can activate the plant’s programmed defense pathways, resulting in reducing the effects of subsequent biotic attack, known as induced systemic resistance (ISR) (Bakker et al., 2013). Actinomycetes, including *Streptomyces* spp., are soil-borne beneficial bacteria that have been shown to trigger ISR *in planta* and inhibit pathogen growth via induction of plant defense mechanisms (Martínez-Hidalgo et al., 2015). Induced systemic resistance induces plant resistance to a broad spectrum of root and foliar pathogens. In this study, we aimed to identify a new antifungal actinomycete from the UAE soil environment; and to determine the effect of the antifungal activity of the metabolites of the promising actinomycete against *T. punctulata in vitro* and *in vivo*. We also compared it to the activity of Cidely^®^ Top, a foliar fungicide treatment, which has been shown to provide protection against black scorch disease on date palms in greenhouse trials. This study has explored the potential to use both biocontrol and fungicides to develop an IDM strategy against this disease.

## Materials and Methods

### Fungal Growth and Disease Assays

The fungus, *T. punctulata* (DSM-102798), was previously identified by Saeed et al. (2016) as the causal agent of the date palm scorch disease in the UAE. *T. punctulata* was cultured on potato dextrose agar (PDA; Lab M Limited, Lancashire, United Kingdom) plates (pH 6.0); supplemented with ampicillin (Sigma–Aldrich Chemie GmbH, Taufkirchen, Germany) used at a rate of 25 mg l^-1^ of agar medium, to inhibit the bacterial contaminants. The pathogen was subcultured on fresh PDA plates every 10 days and incubated at 28°C.

For disease assays, leaf bases of 8-month-old date palm (cv. Chichi) seedlings, obtained from the Date Palm Development Research Unit (DPDRU), United Arab Emirates University, Al-Ain, UAE, were surface-sterilized with 70% ethanol, and mechanical wounding was performed with sterilized scalpels prior to inoculation. These date palm seedlings were inoculated with agar plugs (8-mm in diameter) colonized by fungal mycelium from 10-days old *T. punctulata* cultures at the leaf base region, and the area of inoculation was wrapped with parafilm (Sigma–Aldrich) (Saeed et al., 2016). Inoculated seedlings were maintained in a greenhouse at 28°C, and examined for disease development.

### Isolation and Detection of the Antifungal Activity of Actinomycete Isolates

Five random rhizosphere soil samples limited to 30 cm-depth obtained with a clean spade near the roots of healthy date palm trees, were collected in plastic bags. Rhizosphere soil samples were air-dried for 4 days at 28°C to reduce the numbers of contaminant bacteria (Williams et al., 1972), passed through a 5-mm mesh sieve to remove small stones and root fragments and stored in sterile screw-capped jars at 25°C in the dark, for a week prior to microbiological processing. Actinomycetes were isolated using the soil dilution plate method (Johnson and Curl, 1972) on inorganic salt starch agar (ISSA) (Küster, 1959) amended with cycloheximide and nystatin (each 50 µg ml^-1^; Sigma–Aldrich) with specific soil pre-treatments (Hayakawa and Nonomura, 1987). Briefly, the soil pre-treatments involved preparing serial dilutions of the soil suspension by suspending the sample in 6% yeast extract (YE) (Lab M Limited) and 0.05% sodium dodecyl sulfate (SDS) (Sigma–Aldrich) for 20 min at 40°C, and diluting with water to remove other factors promoting bacterial growth or injurious to germinating actinomycete propagules. The YE and SDS were included to increase and decrease the numbers of actinomycetes and bacteria, respectively (Nonomura and Hayakawa, 1988). Seven replicate plates were used per dilution, which were incubated at 28°C in dark for 7 days. Actinomycete colonies were counted, transferred onto oatmeal agar plates (ISP medium 3) supplemented with 0.1% yeast extract (OMYEA) (Küster, 1959) and stored in 20% glycerol (cryoprotectant) at -20°C (Wellington and Williams, 1978). All isolates were tentatively identified and grouped to the genus level on the basis of their standard morphological criteria and according to the absence or presence of aerial mycelium, distribution (aerial/substrate) and form of any spores present and stability or fragmentation of substrate mycelium (Cross, 1989).

We screened all 47 rhizosphere actinomycete isolates obtained, for their potential to produce diffusible antifungal metabolites active against *T. punctulata* using the cut-plug technique (Pridham et al., 1956). Actinomycete isolates were inoculated on OMYEA plates and incubated at 28°C in dark for 7 days. Plugs were then cut from the growing margins of the actinomycete colonies with a sterilized 11-mm cork-borer and transferred aseptically to the center of PDA plates freshly seeded with *T. punctulata* and further incubated at 28°C in dark for 4 days. The diameters of zones of inhibition were determined in mm. Three replicates were used for each actinomycete isolate.

Inocula for the preparation of the PDA seeded plates were prepared by cultivating *T. punctulata* on PDA slants at 28°C until abundant sporulation occurred. The slant surfaces were then flooded with 50 mM phosphate buffer (pH 6.8), and spores as well as some mycelial fragments were dislodged by scraping the surface with a sterilized scalpel; and were homogenized in an Omni-mixer (OCI Instruments, Omni Corporation International, Waterbury, CT, United States) at 4000 rpm for 20 min. The resultant suspensions were diluted and added to sterile cooled PDA prior to the pouring of the plates. A suspension of approximately 10^8^ CFU ml^-1^ was used as inoculum. The control consisted of PDA plates with OMYEA plugs without any actinomycete growth.

Based on the results obtained from this experiment, only the most promising antagonistic BCA (isolate #7) that produced the strongest inhibition of *T. punctulata* was selected for further analysis. The rest of the isolates were considered either to be non-producers of antifungal metabolites active against *T. punctulata* or those that produced only low or non-detectable levels of inhibition against *T. punctulata*, and were therefore not included in the subsequent studies.

### Identification and Detection of the Antifungal Activity of the Promising BCA

Identification of the BCA isolate #7 to the species level was based on morphological, cultural, and physiological characteristics as described by Locci (1989). Light microscopy (100X) was carried out using Nikon-Eclipse 50i light microscope (Nikon Instruments Inc., Melville, NY, United States), whilst scanning electron microscopy (SEM) was carried out using the Philips XL-30 SEM (FEI Co., Eindhoven, The Netherlands). We further confirmed the identification by 16*S* rRNA gene sequence analysis done by the Deutsche Sammlung von Mikroorganismen und Zellkulturen GmbH, (DSMZ), Braunschweig, Germany. The partial 16*S* rRNA gene sequence (about 1501 nucleotides) was determined by direct sequencing of PCR-amplified 16*S* rRNA as described by Rainey et al. (1996). PCR conditions were: initial denaturation of 3 min at 95°C, followed by 28 cycles of denaturation at 95°C for 1 min, primer annealing at 55°C for 1 min and extension at 72°C for 2 min. A final extension step consisted of 5 min at 72°C was also included. Phylogenetic tree was constructed to predict the species level characterization of the studied isolate using the neighbor-joining method implemented in Molecular Evolutionary Genetics Analysis 7.0 (MEGA7) software (Saitou and Nei, 1987; Kumar et al., 2016).

We also tested the ability of the BCA (isolate #7) for its potential to produce diffusible antifungal metabolites active against *T. punctulata* using the cup plate method (Bacharach and Cuthbertson, 1948). Individual 250 ml Erlenmeyer flasks containing 50 ml of sterile fish meal extract broth (FMEB; El-Tarabily et al., 1997) were inoculated with 1 ml of 10% glycerol suspension of the BCA (approximately 10^8^ CFU ml^-1^) and incubated in a gyratory shaker (Model G76, New Brunswick Scientific-Edison, Edison, NJ, United States) at 200 rpm at 28°C in dark for 5 days. After incubation, the suspensions from each flask were centrifuged for 30 min at 2000 *g*. The supernatant (crude culture filtrate) was filtered through sterile Millipore membranes of pore size 0.22 µm (Millipore Corporation, Billerica, MA, United States) and collected in sterilized tubes and stored at 4°C.

Inocula for the preparation of the *T. punctulata* seeded PDA plates were prepared as described above for the cut-plug technique. Wells were cut in the centers of the fresh PDA plates seeded with *T. punctulata* using a sterilized 11-mm cork-borer. Aliquots (0.3 ml) of the filter-sterilized crude culture filtrate were pipetted into the wells using a sterilized syringe. The plates were incubated at 28°C in dark for 4 days; and the diameters of zones of inhibition were measured in mm. Filter-sterilized inorganic salt starch broth without the BCA was similarly pipetted into the wells in the PDA plates seeded with *T. punctulata* as a control.

In addition, we conducted a dialysis membrane overlay technique to assay inhibition of *T. punctulata* by the BCA. The single thickness dialysis membrane overlay technique (Gibbs, 1967) was used on fish meal extract agar (FMEA; El-Tarabily et al., 1997). The 90-mm dialysis membrane (Type 45311; Union Carbide Corporation, Alsip, IL, United States) was overlaid on FMEA and the membrane surface was inoculated with the BCA by evenly streaking cells and/or spores of a 7-day old culture of the BCA grown on OMYEA. The plates were incubated at 28°C in dark for 10 days. The membranes with the adhering colonies were subsequently removed from the agar plates and the center of each plate was inoculated with a disk (5-mm in diameter) of *T. punctulata* culture grown for 7 days on FMEA. Plates were incubated at 28°C in dark. The colony diameter of *T. punctulata* was measured in mm after 8 days and compared to that of FMEA plates (control) where the pathogen was grown without the BCA. At the end of the incubation period, if the pathogen had not grown from the agar plugs, these plugs were further transferred to a fresh PDA plate and incubated at 28°C for 5 days to determine whether the diffused metabolites were fungicidal (no pathogen growth from the plug) or fungistatic (pathogen growth from the plug).

### Assays of Producing Volatile Antifungal Compounds, Hydrocyanic Acid and Siderophores by the BCA

Production of volatile antifungal metabolites by the BCA was tested on FMEA as described by Payne et al. (2000). Briefly, FMEA plates were inoculated with the BCA by evenly streaking cells and/or spores from a 7-day old culture onto the whole surface of the agar. These cultures were grown at 28°C in dark for 14 days. At this time, plates of the same medium were inoculated with an actively growing *T. punctulata* mycelial plug (5-mm in diameter). The lids were removed and the plates containing the pathogen were inverted over the BCA plates. The two plate bases were taped together with a double layer of Parafilm (American National Can TM, Greenwich, CT, United States). Control plates were prepared in the same way except that a non-inoculated FMEA plate was used instead of a plate containing the BCA. After a further 7 days of incubation, the colony diameter of *T. punctulata* growing in the presence of the BCA was measured and compared to that of the control.

Hydrogen cyanide (hydrocyanic acid) production by the BCA was detected as described by Bakker and Schippers (1987). The change in color from yellow to orange–brown on the filter paper impregnated with 0.5% picric acid and 2% sodium carbonate indicated the production of cyanide.

Plates of chrome azurol S (CAS) agar developed by Schwyn and Neilands (1987) and modified by Alexander and Zuberer (1991) known as modified M9 agar, were inoculated with the BCA and incubated at 28°C in dark for 10 days. Development of yellow–orange halo zone around the culture was considered as positive for siderophore production (Alexander and Zuberer, 1991).

### Determination of Chitinase and β-1,3-glucanase Activities of the BCA

The BCA was inoculated onto a colloidal chitin agar (Gupta et al., 1995) plate and incubated at 28°C in dark until zones of chitin clearing were seen around and beneath the colonies. Colloidal chitin was prepared from crab shell chitin (Sigma–Aldrich) (Hsu and Lockwood, 1975). Clear zone diameters were measured in mm to indicate the chitinase activity of the isolate.

Quantitative production of chitinase and β-1,3-glucanase by the BCA were also determined as described previously (Singh et al., 1999) using the minimal synthetic medium (Tweddell et al., 1994) amended with 2 mg ml^-1^ of either colloidal chitin or laminarin (Sigma–Aldrich), respectively.

Chitinase specific activity was calculated by measuring the release of *N*-acetyl-D-glucosamine (NAGA) from colloidal chitin. Specific activity (U = 1 unit of chitinase) was defined as the amount of the enzyme that released 1 µmol of NAGA mg^-1^ protein h^-1^ (Reissig et al., 1955). The specific activity of β-1,3-glucanase was determined by measuring the amount of reducing sugars liberated from laminarin using dinitrosalicylic acid (DNS) solution (Miller, 1959). Specific activity (U = 1 unit of β-1,3-glucanase) was defined as the amount of the enzyme that released 1 µmol of glucose mg^-1^ protein h^-1^ (Miller, 1959). Protein content of the enzyme solution was determined by the Folin phenol reagent method (Lowry et al., 1951).

### Effect of BCA Crude Culture Filtrate on *Thielaviopsis punctulata* Mycelial Growth, Spore Germination and Germ Tube Elongation

The assay for inhibition of colony and mycelial growth was conducted on PDA plates as described by Lorito et al. (1993). The crude culture filtrate was mixed with sterilized PDA (45°C at 10, 25, 50, 75 and 100% proportions) and poured into Petri plates. Control plates contained 0% crude culture filtrate. The medium was inoculated with a 5-mm in diameter agar plug with actively growing *T. punctulata* mycelium (placed colonized surface down) in the center of the plates. The colony growth of *T. punctulata* was compared with that of the control after 5 days of incubation in the dark at 28°C.

The assay for inhibition of mycelial growth was also conducted in potato dextrose broth (PDB) (Lorito et al., 1993). Prepared crude culture filtrate was mixed with sterilized PDB at 0, 10, 25, 50, 75, and 100% proportions. The PDB was inoculated with a 5-mm diameter agar plug with actively growing *T. punctulata* mycelium. The mycelial dry weight of *T. punctulata* was measured in g after 10 days of incubation in the dark at 28°C.

The effect of the filter-sterilized crude culture filtrate of the BCA on aleuroconidia germination and germ tube elongation of *T. punctulata* was conducted in PDB as described by Lorito et al. (1993). Briefly, the crude culture filtrate was prepared as described above using FMEB. Aliquots (20 µl) of the crude culture filtrate were mixed with 20 µl of spore suspension of *T. punctulata* and 60 µl of PDB. The control consisted of the FMEB without the BCA replacing the crude culture filtrate. The reaction mixture was incubated at 28°C in dark. After 24 h, the percent spore germination and average length of germ tubes in µm were microscopically determined at 40X using a light microscope (Nikon-Eclipse 50i) and compared with the control.

For the effect of the crude culture filtrate of the BCA on hyphal morphology, *T. punctulata* was grown in 100-ml PDB at 28°C in dark for 10 days. The culture broth was removed and the mycelial mats were aseptically washed four times with sterile distilled water (Sneh, 1981). A 100-ml carbon-deficient salt solution was added to the living mycelium of *T. punctulata*. A 50-ml of the filter-sterilized crude culture filtrate of the BCA was also added to the mycelial suspension. The flasks were incubated at 28°C in dark for 4 days, and the culture inspected daily. Controls consisted of 100 ml carbon-deficient salt solution containing *T. punctulata* mycelium incorporated with 50 ml FMEB, without the BCA.

At each sampling, a sub-sample of *T. punctulata* hyphae was retrieved and any subsequent changes in the hyphal morphology observed at 100X using a light microscope (Nikon-Eclipse 50i) connected with Nikon camera (DS – Flic). Three replicates were used at each sampling.

### *In Vitro* Evaluation of the Minimum Effective Concentration of Cidely^®^ Top Fungicide

An *in vitro* evaluation of the fungicide Cidely^®^ Top (difenoconazole and cyflufenamid; Syngenta International AG, Basel, Switzerland) was carried out as previously described (Jonathan et al., 2012). The fungicide, obtained at a dissolved solution of 25, 75, 125, 250, 500, or 1000 ppm final concentration in sterile water, was introduced aseptically into sterilized molten PDA at 25°C. The molten PDA was amended with ampicillin (Sigma–Aldrich) to inhibit bacterial growth. The solution was carefully swirled to attain homogenization status. The resulting mixtures were aseptically dispensed into sterile Petri dishes. A sterile cork-borer measuring 5-mm in diameter was used to introduce the tested pathogen onto the control (without fungicide) and treatment (with fungicide) media. Cultures were incubated at 28°C in dark for 15 days after which radial growth measurements were recorded daily. The percentage of the mycelial growth was measured and growth inhibition was calculated according to the following equation: Mi % = (Mc – Mt)/Mc × 100%; where; Mi, Inhibition of the mycelial growth; Mc, colony diameter (in mm) of control set; and Mt, colony diameter (in mm) of the target fungus on the medium with fungicide.

### *In Vivo* Experiments with the BCA and the Fungicide

*In vivo* evaluations of the BCA and the fungicide were carried out on 8-month-old date palm plants (cv. Chichi). Seedlings were divided into two experiments conducted in parallel:

Experiment I: The purpose of this experiment was to compare the minimum effective dosage concentration of Cidely^®^ Top fungicide with the BCA in controlling black scorch disease. For the fungicide treatment, only one cylindrical wound was made in the leaf base of seedlings to introduce one agar plug of the inoculum. At 2 weeks post inoculation (wpi) (when disease symptoms were evident), we sprayed the inoculated seedlings with 100 ml of Cidely^®^ Top (250 ppm) fungicide, as previously described by Saeed et al. (2016). Healthy controls were wounded and treated with PDA disks; disease controls were wounded and treated with PDA disks colonized with the pathogen. For BCA treatments, three cylindrical wounds (8-mm in diameter) were made in each leaf base of date palm seedlings in order to introduce one agar plug of the *T. punctulata* inoculum at 2 wpi preceding the insertion of two agar plugs of the BCA treatment. Six plants in separate pots were used for each group/treatment; and pots were arranged in a completely randomized design. The plants were grown for another 16 weeks before the number of dead plants in each treatment was counted. The experiment was repeated three times. Groups/treatments for Experiment I were as follows:

(1) Healthy controls (C): Non-inoculated- or non-treated-seedlings;
(1) Diseased controls (*Tp*): Inoculated-seedlings with *T. punctulata* only;
(1) BCA controls (BC): Inoculated-seedlings with BCA only;
(1) Chemical fungicide treatment (*Tp*+CC): Sprayed-seedlings with Cidely^®^ Top 2 weeks after inoculation with *T. punctulata*; and
(1) BCA treatment: Inoculated-seedlings with BCA 2 week after *T. punctulata* inoculation (*Tp*+BC).

Experiment II: The purpose of this experiment was to investigate the effectiveness of various timings of BCA treatment to manage black scorch disease. Similar to the BCA treatment described above, methods of inoculation with *T. punctulata* and BCA application was used. In this experiment, the timing of the BCA treatment was the important factor (preventive, concurrent or curative). The set-up of the treatments/groups for Experiment II was as follows:

(1) Diseased controls (*Tp*): Inoculated-seedlings with *T. punctulata* only;
(1) BCA controls (BC): Inoculated-seedlings with BCA only;
(1) Preventive treatment: Inoculated-seedlings with BCA 1 week before *T. punctulata* inoculation;
(1) Concurrent treatment: Inoculated-seedlings with BCA at the same time of *T. punctulata* inoculation; and
(1) Curative treatment: Inoculated-seedlings with BCA 1 week after *T. punctulata* inoculation.

Six plants in separate pots, arranged in a completely randomized design, were used for each treatment/group. Plants were grown for another 16 weeks before the number of dead plants in each treatment was counted. The experiment was conducted three times.

**FIGURE 1:**
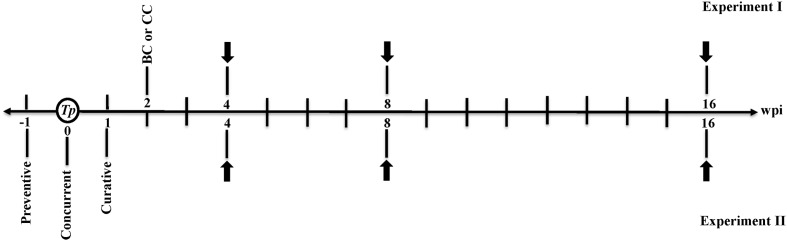
Timeline of microbial inoculation, BCA and fungicide treatments, and disease evaluation schedule. Preventive: BCA treatment 1 week before *Thielaviopsis punctulata* inoculation; Concurrent: BCA treatment at the same time of *T. punctulata* inoculation; Curative: BCA treatment 1 week after *T. punctulata* inoculation; BC or CC: BCA or Cidely^®^ Top fungicide (chemical treatment) at 2 wpi with *T. punctulata*. White circle, establishment of *T. punctulata* disease assay on date palm (cv. Chichi) seedlings; black arrow, disease progress and treatment evaluation at 4, 8, and 16 wpi with *T. punctulata*; *Tp*, *T. punctulata*; CC, chemical control using Cidely^®^ Top fungicide; BC, biocontrol treatment using the BCA candidate (*Streptomyces globosus* UAE1); BCA, biocontrol agent; wpi, weeks post inoculation.

Control and inoculated seedlings were further kept in a greenhouse with a photoperiod extended to 15 h under fluorescent lights (160 W mol^-1^ m^-2^ s^-1^) at 28°C until disease symptoms were evident, and frequent observations were made until 16 wpi with *T. punctulata*. The schedule of the timing of *T. punctulata* inoculation, the actinomycete BCA isolate and chemical fungicide treatments, and disease evaluation are presented in **Figure 1**.

### Spore Counts and Disease Severity Index in Inoculated Plants

The growth of *T. punctulata* in inoculated plants was determined on the basis of the number of fungal spores (total spore counts) at 16 wpi. Three tender leaf bases from six inoculated seedlings per treatment were collected, cut into small pieces (2–5 mm in diameter), soaked in 10 ml distilled water and vigorously agitated for 30 min. Harvested spores were counted using haemocytometer (Agar Scientific Limited, Essex, United Kingdom).

Disease severity index (DSI) was recorded at 8 and 16 wpi using a scale of 0–5: 0 = no apparent symptoms, 1 = 1–10% necrotic or dark brown area around the point of infection, 2 = 11–25%, 3 = 26–50%, 4 = 51–75%, and 5 = 76–100% (Molan et al., 2004). All experiments were repeated three times with similar results.

### Statistical Analysis

For the *in vitro* evaluation of Cidely^®^ Top fungicide and BCA against *T. punctulata*, data were analyzed using the analysis of variance (ANOVA) while means were separated using Duncan’s multiple range test at 5% level of significance. These experiments were repeated in triplicates using five plates/treatment for each time with similar results.

For the fungal spore counts and DSI of the *in vivo* treatments against *T. punctulata*, three replicates for each group were examined. Data represent the mean ± SD from a minimum of six plants. ANOVA and Duncan’s multiple range test were performed to determine the statistical significance at *P* < 0.05. Similar results were obtained in each replicate. SAS Software version 9 was used for all statistical analyses performed (SAS Institute, 2002).

## Results

### Isolation, Identification and Screening of Actinomycetes Isolates

The population of actinomycetes in the date palm rhizosphere was found to be 6.73 ± SE 1.28 log_10_ CFU g^-1^ dry soil. Forty-seven streptomycete and non-streptomycete actinomycete strains were isolated, of which 37 isolates (78.8%) belonged to the genus *Streptomyces*, whilst only 10 isolates (21.2%) belonged to genera of non-streptomycete actinomycetes. The latter isolates were found to be affiliated to the genera *Micromonospora*, *Rhodococcus*, *Streptoverticillium*, and *Nocardia*. We also found that 25.5% (12/47) of the rhizosphere actinomycete isolates (9 streptomycete and 3 non-streptomycete) produced strong diffusible antifungal metabolites active against *T. punctulata* using the cut-plug technique (**Table 1**). All the 12 isolates produced large zones of inhibition (>20 mm) against *T. punctulata*. The remaining isolates were either non-producers of diffusible antifungal metabolites active against *T. punctulata* or produced very low levels of inhibition against *T. punctulata* (inhibition zones < 20 mm); they were therefore not included in the subsequent studies. Based on the results obtained, the most promising inhibitory and antagonistic BCA candidate (isolate #7) producing the strongest inhibition against *T. punctulata* was selected for further analyses (**Table 1**; Supplementary Figure S1).

**Table 1:** Diameter of the inhibition zone of tested actinomycete isolates against *Thielaviopsis punctulata* using the cut-plug technique.

Actinomycete		Diameter of inhibition zone (mm)^a^
	
Genera	Isolate	
*Streptomyces* sp.	#3	22.50 ± 0.64 *fg*
	#7 (BCA)	33.80 ± 0.44 *a*
	#10	26.00 ± 0.70 *e*
	#18	21.62 ± 0.74 *g*
	#21	28.87 ± 0.42 *cd*
	#23	30.57 ± 0.44 *b*
	#33	29.05 ± 0.41 *cd*
	#38	23.57 ± 0.44 *f*
	#45	31.27 ± 0.32 *b*
*Micromonospora* sp.	#15	29.87 ± 0.42 *bc*
*Streptoverticillium* sp.	#29	25.37 ± 0.34 *e*
*Rhodococcus* sp.	#42	28.25 ± 0.41 *d*


*^a^Values are means of three replicates ± SE. Values with the same letter are not significantly different at *P* = 0.05. Isolate #7 represents the biocontrol agent (BCA), *Streptomyces globosus* UAE1.*

### Identification of the BCA Isolate #7 to the Species Level

**FIGURE 2:**
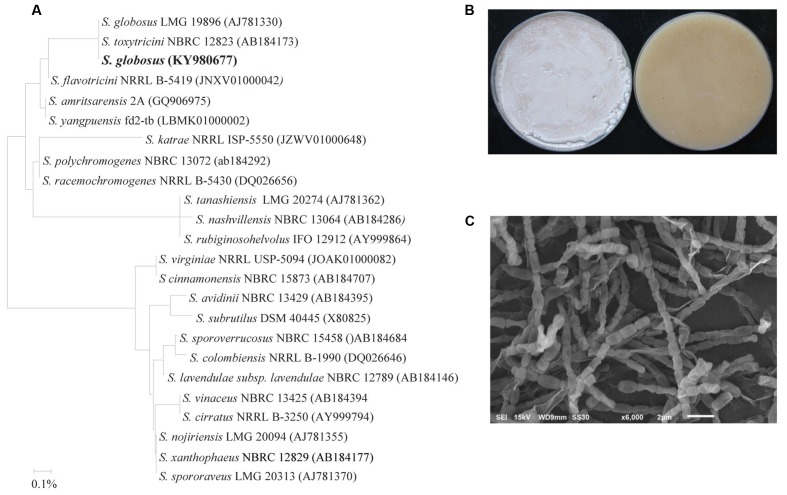
Identification of *Streptomyces globosus* UAE1 based on phylogenetic, cultural and morphological characteristics. **(A)** The dendrogram showing the phylogenetic relationships between *S. globosus* UAE1 (KY980677) and other members of *Streptomyces* spp. on the basis of 16*S* rRNA sequences prepared by the neighbor-joining method. **(B)** Beige aerial mycelium (left) and yellow substrate mycelium (right) growing on (ISP medium 3) supplemented with yeast extract, and **(C)** Scanning electron micrograph (6,000X) of the Rectiflexibiles spore chains and smooth-surfaced spores of *S. globosus* UAE1. In **(A)** the scale bar on the rooted tree indicates a 0.01 substitution per nucleotide position.

The potential antagonistic BCA candidate (isolate #7) was isolated and preliminarily identified by nucleotide sequence of its 16*S* rRNA gene. The resulting sequence data from this strain was deposited in NCBI (GenBank Accession Number: KY980677). In addition, a comparison with representative 16*S* rRNA gene sequences of organisms belonging to the Actinobacteria was carried out using phylogenetic analysis. Comparison of the 16*S* rRNA gene of isolate #7 (∼1501 bp) with sequences in the GenBank database revealed that the BCA candidate was a streptomycete sp. with 100% similarity to *Streptomyces globosus* (AJ781330) and *S. toxytricini* (AB184173) (**Figure 2A**). The rest of the *Streptomyces* spp. showed less than 99.7% similarity with the target antagonistic strain. This suggests that the isolated strain could possibly be either *S. globosus* or *S. toxytricini*; thus, it was necessary to obtain a more definitive identification of the isolate.

To confirm the species of isolate #7, pure cultures were cultivated on ISP medium 3 supplemented with yeast extract (OMYEA). Cultures typical white, turning to beige, aerial mycelium and yellow substrate mycelium were evident after 14 days of cultivation (**Figure 2B**). Using SEM, the configuration of the spore chains of the isolate revealed spore chain morphology which belongs to Rectiflexibiles (RF) form, consisting of cylindric or elongated smooth spores (0.75 × 1.0 µm) on an aerial mycelium (**Figure 2C**). Together, this suggests that the outstanding isolate #7 can be identified as *S. globosus* (Krassilnikov, 1941) Waksman in Waksman and Lechevalier (1953) Strain UAE1.

### *In Vitro* Evaluation of Antagonistic Properties of the BCA Isolate

**FIGURE 3:**
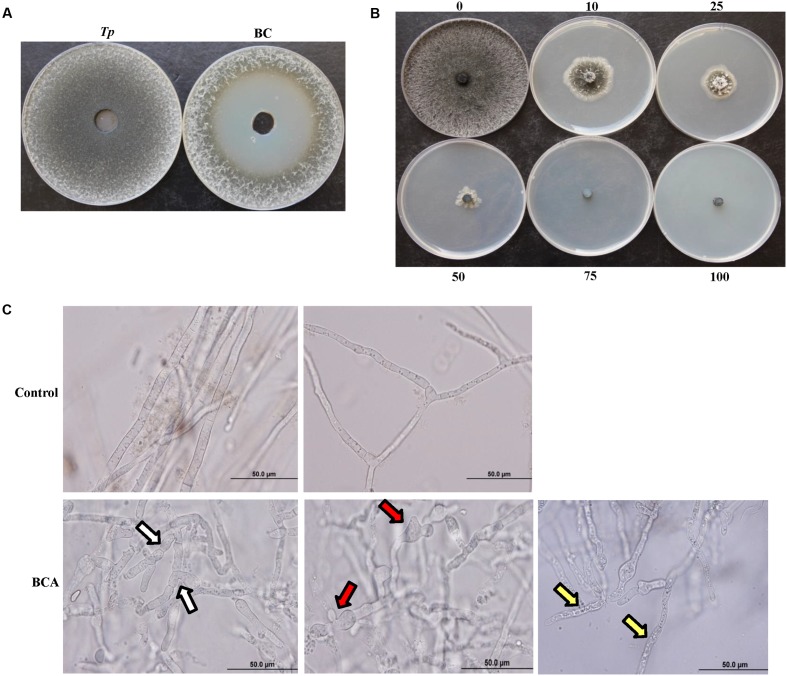
Inhibitory effect of the BCA candidate on *Thielaviopsis punctulata*. **(A)** Inhibition of *T. punctulata* mycelial growth by *Streptomyces globosus* UAE1 (isolate #7) using the cup plate technique, **(B)** Gradual inhibition of *T. punctulata* colony growth on PDA plates containing different proportions (%) of crude culture filtrate of *Streptomyces globosus* UAE1 (isolate #7), and **(C)** Abnormalities evident in hyphal morphology and cytoplasmic contents of *T. punctulata*, following exposure to *S. globosus* UAE1 (isolate #7) metabolites (bottom panel), compared to control (top panel). White arrows point to hyphal septum malformation and branch deformation; while red and yellow arrows point to hyphal swellings and cytoplasmic coagulation, respectively.

The incorporation of the filter-sterilized crude culture filtrate of the BCA into the wells using the cup-plate technique resulted in significant (*P* < 0.05) retardation of the growth of *T. punctulata*, producing large zones of inhibition (45 mm ± SE 0.88), caused by the diffused antifungal metabolites, when compared to the control treatment (**Figure 3A**). Following the removal of the dialysis membranes from the FMEA, the growth of the inoculum of *T. punctulata* was clearly inhibited by the diffused metabolites of the BCA, when compared to the control or the non-diffusible antifungal metabolite-producing *Streptomyces* sp. (isolate #25) (Supplementary Figure S1). The pathogen did not grow from the plugs transferred from treatment plates to the fresh PDA medium in the absence of diffused metabolites. This confirmed that the BCA showed fungicidal activities to *T. punctulata*.

In order to determine whether the BCA produced volatile antifungal compounds, isolate #7 along with the positive (isolate #25) and negative controls were grown on FMEA. Similar to the control which did not contain any actinomycete isolates, the growth of *T. punctulata* over the BCA was not affected with any visible effect on colony morphology when compared to the volatile antifungal producing *Streptomyces* (isolate #25) which caused total inhibition of the growth of the pathogen (Supplementary Figure S2). The BCA also failed to produce hydrogen cyanide or siderophores. The BCA did not produce clear inhibition zones around or beneath the colonies when grown on colloidal chitin agar, indicating of the failure of the tested BCA to produce chitinase compared to the chitinase-producing *Micromonospora* sp. (isolate #4) (Supplementary Figure S2). In addition, the BCA did not produce any detectable levels of chitinase or β-1,3-glucanase, when grown in the liquid medium containing colloidal chitin or laminarin, respectively. Together, our data suggest that the inhibition of the causal agent of black scorch is the result of the activities of the diffusible antifungal metabolites of the BCA.

### Inhibition of *Thielaviopsis punctulata* Mycelial Growth and Spore Germination by the Crude Culture Filtrate of the BCA

We demonstrated that the culture filtrates of the BCA were effective in inhibiting growth of *T. punctulata*. On PDA plates, the increasing levels of the BCA culture filtrates increasingly and significantly inhibited the colony and mycelial growth of *T. punctulata* after 5 days of incubation at 28°C (**Figure 3B** and **Table 2**). A total inhibition of *T. punctulata* mycelial growth was observed when culture filtrates were supplied with 75% or above. In PDB, the assay for inhibition of mycelial growth showed a similar trend to the effect of the BCA filter-sterilized crude culture filtrate on the mycelial growth of the pathogen on PDA plates. The culture filtrates of the BCA significantly inhibited the mycelial growth of *T. punctulata* when incorporated into PDB with increasing proportions compared to the control after 5 days of incubation at 28°C (**Table 2**). Moreover, the germination of aleuroconidia and the average length of germ tubes produced by *T. punctulata* were significantly reduced in the presence of the filter-sterilized crude culture filtrate of the BCA after 24 h of incubation compared with those without BCA (**Table 2**). This suggests that the culture filtrates that supported the BCA not only inhibited spore germination and germ tube elongation, but also mycelial growth of *T. punctulata in vitro*.

**Table 2:** Inhibition of *Thielaviopsis punctulata* mycelial growth, spore germination and germ tube elongation by the crude culture filtrate of the BCA.

Culture filtrate (%)	Colony diameter (mm)^a^	Mycelial dryweight (g)^a^	Spore germination(%)^a^	Germ tube length (µm)^a^
0	99.03 ± 0.28*^a^*	70.28 ± 2.94*^a^*	88.11 ± 1.98*^a^*	54.66 ± 1.54*^a^*
10	39.63 ± 1.68*^b^*	39.36 ± 2.79*^b^*	32.88 ± 1.52*^b^*	40.65 ± 2.21*^b^*
25	22.08 ± 1.34*^c^*	18.93 ± 1.49*^c^*	19.04 ± 1.69*^c^*	21.83 ± 1.64*^c^*
50	13.03 ± 0.09*^d^*	9.35 ± 1.19*^d^*	9.16 ± 1.01*^d^*	12.01 ± 1.06*^d^*
75	0.00 ± 0.00*^e^*	1.98 ± 0.18*^e^*	3.95 ± 0.51*^e^*	7.64 ± 1.05*^e^*
100	0.00 ± 0.00*^e^*	0.21 ± 0.10*^e^*	0.30 ± 0.14*^e^*	0.49 ± 0.20*^f^*


*^a^Values are means of three replicates ± SE. Values with the same letter within a column are not significantly different at *P* = 0.05.*

There was also noticeable hyphal abnormalities, including hyphal swelling (ballooning), septum malformation and abnormal branch formation in *T. punctulata* treated with the filter-sterilized crude culture filtrate of the BCA (**Figure 3C**). In addition, the hyphal cells underwent cytoplasmic coagulation in crude BCA culture filtrate-treated flasks. Mycelial mats in control flasks remained healthy and intact.

### *In Vitro* Inhibitory Effect of the Fungicide Treatment on Mycelial Growth of *Thielaviopsis punctulata*

**FIGURE 4:**
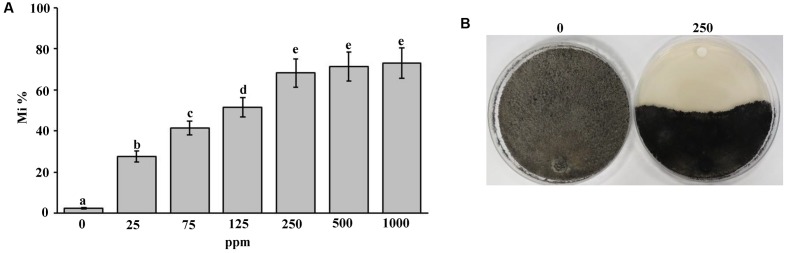
Sensitivity of *Thielaviopsis punctulata* to Cidely^®^ Top fungicide. **(A)** Growth inhibition rate (Mi %) of *T. punctulata* using different concentrations of Cidely^®^ Top after 15 days. Values with different letters are significantly different at *P* = 0.05, and **(B)** Effect of Cidely^®^ Top (250 ppm) on *in vitro* mycelial growth.

In order to evaluate the effect of the fungicide Cidely^®^ Top (difenoconazole and cyflufenamid) to inhibit the mycelial growth of *T. punctulata*, six concentrations (25, 75, 125, 250, 500, or 1000 ppm) of the selected fungicide were applied *in vitro* (Supplementary Figure S3). The data obtained from this study revealed that up to 250 ppm of the fungicide tested; there were significant differences associated with the concentrations applied, in inhibiting the mycelial growth of the pathogen (**Figure 4A**). A significantly increased fungal inhibition zone was evident at the 250 ppm concentration (**Figure 4B**) as well as at the other two high concentrations tested (500 or 1000 ppm), ranging between 68 and 73% mycelial growth inhibition (stationary growth state). Thus, there were no significant differences between the 250 ppm and higher concentrations of treatments (**Figure 4A**). This suggests that the systemic fungicide (Cidely^®^ Top) was adequately successful in inhibiting the mycelial growth of *T. punctulata*, at 250 ppm and therefore is considered to be the most appropriate fungicide concentration to serve as a minimum inhibitory dose for used in greenhouse or field trials.

### Effect of the Minimum Dose of the Fungicide (Cidely^®^ Top) and BCA on Mycelial Growth of *Thielaviopsis punctulata*

**FIGURE 5:**
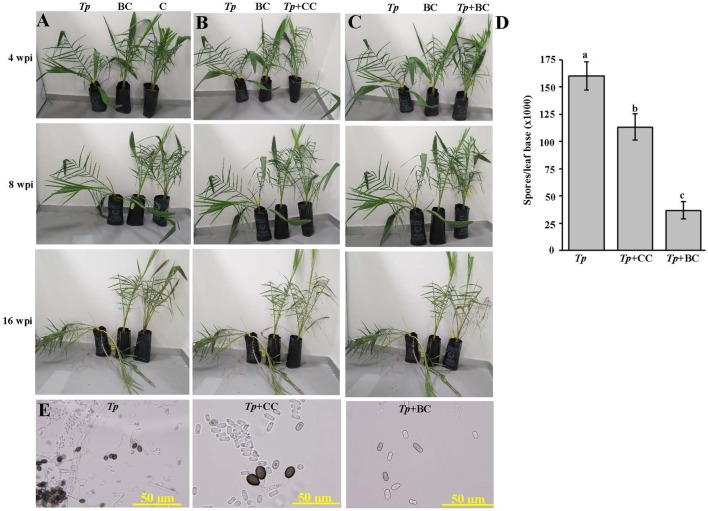
Effect of the biocontrol agent and Cidely^®^ Top fungicide on *Thielaviopsis punctulata*-infected plants. **(A)** Pathogenicity test on *T. punctulata*-inoculated date palm seedlings (*Tp*). Effect of **(B)** Cidely^®^ Top fungicide treatment after *T. punctulata* inoculation (CC + *Tp*), and **(C)** biocontrol treatment in controlling black scorch disease on date palm (BC + *Tp*) at 4, 8, and 16 wpi on *T. punctulata*-inoculated seedlings. **(D)** Number of spores ml^-1^ at 16 wpi after inoculation with *T. punctulata*. Data for spores ml^-1^ represent the mean ± SE from a minimum of 12 inoculated regions; and values with different letters are significantly different at *P* = 0.05. **(E)** Pathogen conidia reisolation from affected tissues of the sick *T. punctulata*-inoculated seedlings (left), and the recovering fungicide- and BC-treated plants. In **(B–E)** seedlings inoculated with *T. punctulata* at 2 weeks before the fungicide or biocontrol treatment. Experiments were repeated at least three times with similar results. *Tp*, inoculated-seedlings with *T. punctulata* only; BC, inoculated-seedlings with BCA only; C, control (no inoculation or treatment); *Tp* + BC, inoculated-seedlings with BCA 2 weeks after *T. punctulata* inoculation, *Tp* + CC, sprayed-seedlings with the chemical control (Cidely^®^ Top) 2 weeks after inoculation with *T. punctulata*; wpi, weeks post inoculation.

A previous study detailed the disease symptoms and the identity of the causal pathogen of the black scorch disease on date palm in the UAE (Saeed et al., 2016). The responses of the pathogen *in vitro* clearly indicated the BCA and the minimum dosage concentration (250 ppm) of Cidely^®^ Top fungicide to be effective against date palm black scorch caused by *T. punctulata*. In order to evaluate the chemical Cidely^®^ Top fungicide as a possible IDM component, and compare that with the BCA in suppressing *T. punctulata*, an *in vivo* experiment was conducted in the greenhouse (Experiment I). Firstly, a pathogenicity test was done to determine the effect of *T. punctulata* on date palm seedlings. Typical symptoms of black scorch disease after 4 wpi with *T. punctulata* (*Tp*) were observed (**Figure 5A**). The disease progressed with time and leaves of infected seedlings showed distinct bending at 16 wpi. No disease symptoms were noticed in inoculated BCA alone (BC) or non-inoculated seedlings (C) (**Figure 5A**). Secondly, we sprayed Cidely^®^ Top on diseased seedlings 2 weeks after inoculation with *T. punctulata* (CC + *Tp*) and assessed the efficacy of the fungicide for another 14 weeks post treatment (wpt; corresponding to 16 wpi with *T. punctulata*). Since we did not find major differences in the inhibition zone of mycelial growth at higher concentrations of Cidely^®^ Top fungicide *in vitro*, we used only 250 ppm (the minimum effective concentration) in the greenhouse. At 4 wpi, the inoculated plants treated with the fungicide (CC + *Tp*) started to recover, which was in contrast to *T. punctulata*-inoculated plants (*Tp*) (**Figure 5B**). We also observed that fresh leaves emerged from the heart of date palm of inoculated seedlings treated with Cidely^®^ Top at 8 wpi, and all dried leaves dropped by 16 wpi (**Figure 5B**). This confirmed our *in vitro* results of the inhibitory effect of the lowest effective dose of Cidely^®^ Top on mycelial growth of *T. punctulata*. Thirdly, we applied the actinomycete BCA on seedlings after 2 wpi with *T. punctulata* (BC + *Tp*). Plants inoculated with the BCA candidate following inoculation with *T. punctulata*, recovered when compared with seedlings inoculated with *T. punctulata* (*Tp*) at all time points of inoculation with the pathogen, and appeared to be healthy and were comparable to plants that were inoculated with BCA alone (BC) (**Figure 5C**). This suggests that this BCA candidate also effectively inhibits *T. punctulata* growth *in vivo*.

We also compared the responses of the pathogen to the fungicide and the biological treatments to determine their effects on spore numbers and conidial morphology. Therefore, the spore counts at leaf base of treated date palm plants were determined. The BCA candidate (BC + *Tp*) caused a greater reduction of the number of spores, followed by Cidely^®^ Top-treated plants (CC + *Tp*) (**Figure 5D**). At least a three-fold reduction in total spore numbers of *T. punctulata* in BCA-treated seedlings were observed compared with the fungicidal treatment and was also associated with the absence of the thick-walled, dark brown and oval aleuroconidia (chlamydospores) (**Figure 5E**). Only the second type of smooth-walled, hyaline and cylindrical phialoconidia (endoconidia) was observed in the BCA treatment (BC + *Tp*); even these occurred in lesser amount than in *T. punctulata*-inoculated (*Tp*) or fungicide-treated seedlings (CC + *Tp*) (**Figure 5E**). In general, the pathogen appeared not to be adequately aggressive to support disease progression when BCA was applied, while only a moderate inhibitory effect was observed in the case of the Cidely^®^ Top treatment.

### Effect of Application Timing of the BCA on the Pathogenicity of *Thielaviopsis punctulata*

**FIGURE 6:**
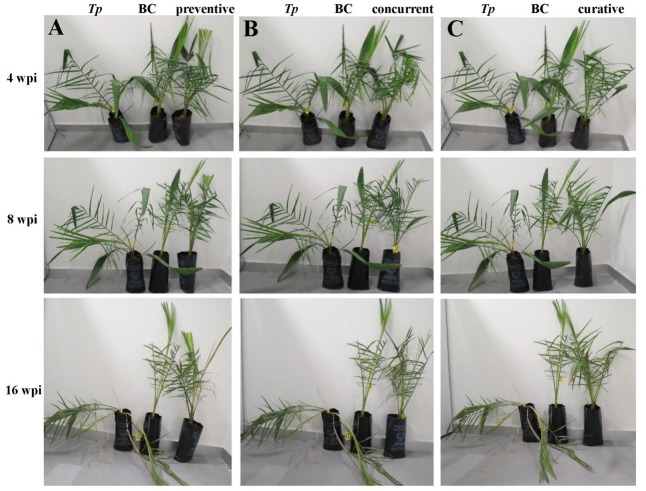
Antagonistic effect of the biocontrol agent against *Thielaviopsis punctulata*. Effect of **(A)** Preventive, **(B)** Concurrent, and **(C)** Curative biocontrol treatment on date palm (cv. Chichi) at 4, 8, and 16 wpi with *T. punctulata.* inoculated-seedlings with *T. punctulata* only; BC, inoculated-seedlings with BCA only; preventive, inoculated-seedlings with BCA 1 week before *T. punctulata* inoculation; concurrent, inoculated-seedlings with BCA at the same time of *T. punctulata* inoculation; curative, inoculated-seedlings with BCA 1 week after *T. punctulata* inoculation. wpi, weeks post inoculation.

In parallel to experiment (I), we evaluated the impact of timing of the application of the BCA on the aggressiveness of *T. punctulata*. For this purpose, three timing intervals of the BCA treatment (Experiment II) were applied to determine the best time management of the BCA on date palm black scorch. They were: (i) 1 week before inoculation with *T. punctulata* (preventive; **Figure 6A**); (ii) at the same time of inoculation (concurrent; **Figure 6B**); or (iii) 1 week after inoculation with the pathogen (curative; **Figure 6C**). All application treatments of the BCA tested suppressed the black scorch disease to varying degrees albeit their timing (**Figure 6**).

We also found significant differences between all treatments (Experiments I and II) when the DSI was calculated. As expected, plants infected with *T. punctulata* (*Tp*) progressed with disease from 8 wpi until they eventually died at 16 wpi (**Table 3**). There was a drastic decrease in DSI in the Cidely^®^ Top-treated seedlings (CC + *Tp*) between 8 and 16 wpi, when compared with that of the same plants inoculated with the pathogen but with no fungicide treatment (*Tp*). Although the two curative BCA treatments at 1 wpi (curative) and 2 wpi (BC+*Tp*) with *T. punctulata* did not show significant difference in the DSI measurements between each other and the fungicide-treated plants, the results obtained from the concurrent application were clearly distinguishable. It is evident that the DSI of concurrent application of BCA and *T. punctulata* was significantly lower than that of the fungicide treatment or any of the BCA curative treatments. In comparison with the *T. punctulata*-infected plants, the DSI of the preventive applications dropped from 4.63 to 2.25 at 8 wpi and 4.88 to 1.88 at 16 wpi, providing 45–65% reduction in disease development. Control (C) seedlings not inoculated with *T. punctulata*, persistently showed no disease symptoms at any time tested. In general, the preventive application a week before inoculation with *T. punctulata* was the most effective treatment in suppressing the pathogen invasion, followed by the concurrent, and then by any of the BCA curative or fungicide treatment. Our data also clearly suggest that the appropriate timing of application of the BCA isolate is a critical factor and should precede *T. punctulata* infection for best results.

**Table 3:** Disease severity index (DSI) of *Thielaviopsis punctulata*-inoculated-date palm seedlings (cv. Chichi) treated with fungicide or BCA (*Streptomyces globosus* UAE1) at 8 and 16 wpi (*n* = 6).

Treatment			DSI^a^
		
		8 wpi	16 wpi
*Tp*		4.63 *a*	4.88 *a*
**Experiment I**	CC + *Tp*	3.50 *b*	3.25 *bc*
	BC + *Tp*	3.00 *bc*	2.88 *c*
**Experiment II**	Curative	3.00 *bc*	2.50 *cd*
	Concurrent	2.75 *c*	2.00 *d*
	Preventive	2.25 *d*	1.88 *e*
BC		0.00 *f*	0.00 *f*
C		0.00 *f*	0.00 *f*


**^a^*DSI is on a scale of 5: 0 = no infection, 1 = 1–10%, 2 = 11–25%, 3 = 26–50%, 4 = 51–75%, and 5 = 76–100% damage necrotic or dark brown area around the point of inoculation. Values with similar letters are not significantly different at *P* = 0.05.*

**Tp*, inoculated-seedlings with *T. punctulata* only; *Tp* + CC, sprayed-seedlings with Cidely^®^ Top 2 weeks after inoculation with *T. punctulata*; *Tp* + BC, inoculated-seedlings with biocontrol agent (BCA) 2 week after *T. punctulata* inoculation; curative, inoculated-seedlings with BCA 1 week after *T. punctulata* inoculation; concurrent, inoculated-seedlings with BCA at the same time of *T. punctulata* inoculation; preventive, inoculated-seedlings with BCA 1 week before *T. punctulata* inoculation; BC, inoculated-seedlings with BCA only; C, control (non-inoculated; non-treated seedlings). wpi, weeks post inoculation.*

## Discussion

Date palm (*Phoenix dactylifera* L.) is a popular crop in the semi-arid and arid regions. Harsh environmental (abiotic) conditions affect plant growth and production of date palm. In addition, organismal (biotic) challenges are significant limiting factors for date yields during critical developmental stages. *T. punctulata*, the main fungal pathogen causing black scorch disease on date palm, has been recorded in various date growing regions in the Arabian Peninsula (Al-Sadi et al., 2012; Al-Naemi et al., 2014) including the UAE (Saeed et al., 2016). The fungus infects the outside leaves, and rapidly kills the younger leaves and the terminal buds (Suleman et al., 2001; Zaid et al., 2002; Abbas and Abdulla, 2003). Therefore, there is an urgent need to develop novel methods to control this disease. In our efforts to develop environmentally sustainable solutions to combat this damaging disease in the UAE, we aimed to isolate an actinomycete strain from the date palm rhizosphere in the UAE; and proceeded to determine its antifungal activity against *T. punctulata*. Moreover, application of fungicides was used as a comparison and also as a part of the IDM strategy to protect plantations against serious invasions by the fungal pathogen.

Fungicides often have “curative” properties, which means they are capable of suppressing the invasion by the pathogen of the host, post-infection. Despite this ability, these chemicals can be active against a pathogen within a few days of infection. In an attempt to search for a successful fungicide for sustained inhibition of *T. punctulata*, we selected Cidely^®^ Top fungicide and tested its efficacy under different conditions. This systemic fungicide was effective in inhibiting the fungus at the tested minimum effective dosage concentration (250 ppm) both *in vitro* and *in vivo*. Previously, we found that three systemic fungicides (Score^®^, Phyton^®^, and Naturame^®^) also reduced mycelial growth *in vitro*; although contact fungicides such as Ortiva^®^ failed to have an impact on fungal growth (Saeed et al., 2016). Similar to Score^®^, the chemical-based fungicide Cidely^®^ Top contains difenoconazole. This indicates that fungicides containing the active ingredient difenoconazole are highly effective in inhibiting mycelial growth of *T. punctulata*. The results obtained from difenoconazole (Score^®^ and Cidely^®^ Top) seem to differ with certain other aspects. A previous report indicated that this fungicide did not stimulate seed germination in sugarcane infected with *T. paradoxa* (Croft, 1998), may be attributable to the different fungicide application methods, plant species or strain differences. Foliar application of Cidely^®^ Top also reduced the number of spores produced by *T. punctulata* in the greenhouse experiment (**Figure 5**) which may be related to the significant reduction in disease symptoms and DSI in the chemical-treated seedlings after 8–16 wpi. Hence, these findings supported our hypothesis that Cidely^®^ Top may serve as a suitable candidate for consideration as a fungicide and a potential method in the IDM strategy against *T. punctulata*.

For decades, control and management of fungal plant diseases have been dependent on the synthetic fungicides. However, frequent use of such fungicides may cause accumulation of toxic compounds potentially hazardous to humans and the environment, and in addition may result in a high risk of pathogens developing resistance to the fungicide. Pretty and Bharucha (2015) have reported that results obtained from IDM approaches by lowering fungicide use will benefit not only farmers, but also global environment and human health. In India, it was found that the most effective component to integrate IDM and control powdery mildew caused by *Leveillula taurica* was to minimize the use of fungicides, resulting in significant yield increase of bell pepper (Kumar et al., 2008). In addition, elimination of early insecticide sprays in irrigated rice areas in Vietnam, saved farmers money and reduced pesticide use (Price, 2001). These are some successful examples of the benefits of adopting this IDM of minimizing chemical applications, and supports our finding of the potential use of the minimal effective spray of Cidely^®^ Top as a component of IDM. Policies, laws and regulations, through authorized agencies or even governments, shall provoke the implementation of IDM to minimize reliance on fungicides, prompting European Union to intervene and promote IDM (Pretty and Bharucha, 2015).

To eliminate the judicious use of these “risky” chemicals, we evaluated the efficacy of actinomycete strains isolated from healthy date palm habitats to inhibit the phytopathogen *T. punctulata*, *in vitro* and *in vivo*. In this study, over 75% of the isolated actinomycetes from the rhizosphere soil of healthy date palms belong to the genus *Streptomyces*. This study is in accordance with the previous reports that streptomycete actinomycetes are known to be predominant among actinomycetes on isolation plates and commonly produce useful antibiotics (∼80% of the total antibiotic production) and active secondary metabolites (Thenmozhi and Krishnan, 2011). The identity of the BCA (isolate #7) was further confirmed by the ribosomal gene (16*S* rRNA) sequence analysis, and the isolate revealed 100% sequence similarity with *S. globosus* (Krassilnikov, 1941) Waksman in Waksman and Lechevalier (1953) and *Streptomyces toxytricini* (Preobrazhenskaya and Sveshnikova, 1957; Pridham et al., 1958). The 16*S* rRNA sequencing has been used as a basic approach for the identification of microbial communities as well as for assessing microbial diversity in natural environments (Solanki et al., 2014). Based on morphological, cultural and physiological characterizations, our results confirmed that the selected species was *S. globosus* (Strain UAE1).

Emphasis was made to look for promising BCAs among actinomycetes as they are known to be relatively suited to be active in dry hot environments such as those in the UAE. They are also known to include many strains capable of functioning as BCAs (El-Tarabily and Sivasithamparam, 2006). *S. globosus* UAE1 exhibited strong antifungal activity against *T. punctulata*, mainly attributable to the production of diffusible antifungal metabolites, but not mycolytic cell-wall degrading enzymes, volatile metabolites, hydrocyanic acid, or iron-chelating siderophores. Multiple dual-culture assays (Bacharach and Cuthbertson, 1948; Pridham et al., 1956; El-Tarabily et al., 1997, 2009) were used in the current study in order to evaluate the nature of the antagonistic activity of this BCA against the black scorch causing agent *in vitro*. In this study, microscopic examination was performed to find out the mode of action and interaction of the antagonistic isolate with the pathogen. The observations revealed that the strain *S. globosus* was capable of causing considerable morphological alternations of hyphae such as cytoplasmic coagulation, shriveled and swelling mycelia, and septal malformations. Similar morphological changes in hyphae due to the activity of antifungal compounds have been demonstrated with other phytopathogens (Wang et al., 2010; Lu et al., 2013). Although many researches have noted promising results relating to microbial antagonism using actinomycetes under laboratory conditions, many of those isolates failed to repeat their performance under greenhouse and field conditions (Doumbou et al., 2001; El-Tarabily and Sivasithamparam, 2006).

To eliminate the discrepancies between *in vitro* and *in vivo* assays, we tested the effect of the strain *S. globosus* UAE1 on *T. punctulata* under greenhouse conditions. In general, our results demonstrated that biological control was more effective in reducing disease development than the chemical fungicide; and this reduction was dependent on the time of application. For example, curative treatments using BCA to established infections by the pathogen would limit damage to the tree and prevent *T. punctulata* spores from germinating within the date palm grove (**Figure 5**). Competition between the BCA and *T. punctulata* applied together led to a greater control efficacy, evident in reduced DSI. We argue that prevention of infection is the best management strategy when it comes to dealing with black scorch disease on date palm plantations. Application of BCA in advance pathogen invasion had more profound effects of biocontrol efficacy, as the main mechanism of protection appears to help establish the required biomass of the BCA ahead of the pathogen invasion and prevent the systemic ingression of *T. punctulata* within the host. The diffusible compounds produced by the BCA candidate were clearly related to the inhibition, destruction and suppression of the invading pathogen within the plant host. There is, however, a possibility of the production by the BCA of compounds capable of inducing host resistance to the pathogen. This possibility was not explored at this stage but certainly will be investigated in future studies. One should not eliminate the possibility that the “preventive” treatment may also induce ISR in plants to manage the black scorch disease on date palm. This form of resistance by the BCA candidate, can be brought through fortifying the physical and mechanical strength of cell wall (Knoester et al., 1999), leading to the synthesis of defense chemicals against *T. punctulata* attack. Defense reactions may activate a diverse array of plant defense genes encoding pathogenesis-related (PR) proteins i.e., chitinase, β-1,3-glucanases, chalcone synthase, phenylalanine ammonia lyase, peroxidase and phytoalexins (Ahn et al., 2002).

The commercial product, Mycostop^®^, is a biological fungicide that contains spores and mycelium from *Streptomyces griseoviridis*. Application of Mycostop^®^ to the root zone of crop plantations, including date palm, reduced spore germination and inhibited hyphal growth of plant pathogenic fungi including *T. punctulata* (Suleman et al., 2002; Minuto et al., 2006). Other commercial *Streptomyces* biocontrol agents, such as *Streptomyces lydicus* (Actinovate^®^, Micro108^®^ or Actino-iron^®^) and *Streptomyces saraceticus* (YAN TEN), have been released to the market (Elliott et al., 2009; Palaniyandi et al., 2013). This suggests that the *Streptomyces* strain isolated in this study can serve as a potential antifungal product against *T. punctulata*. Whilst a number of recent reports have focused on biological controls using species of *Trichoderma* or *Chaetomium* against *T. punctulata* or *T. paradoxa* growth (Soytong et al., 2005; Chakrabarty et al., 2013; Al-Naemi et al., 2016), the current study demonstrates, for the first time, the feasibility of using a streptomycete actinomycete isolate as a BCA against black scorch disease caused by *T. punctulata*.

Application of a BCA can be considered as a successful practice only if it is relatively safe to humans (U. S. Environmental Protection Agency, 2005), is effective over a long duration (Clewley et al., 2012), survives under adverse conditions (McFadyen, 1998) and, if possible, improves plant growth (Mefteh et al., 2017). *Streptomyces* spp. are inexpensive, long lasting, safe, and can survive various harsh conditions (Ningthoujam et al., 2009). Similarly, the isolated *S. globosus* UAE1 strain has the capability to produce spores under extreme heat and drought conditions common to the UAE environment. In this study, actinomycetes were specifically targeted because they are likely better adapted to the UAE environment compared to other bacteria or fungi; in addition of having the ability to be active under conditions prevalent in the dry and arid environments (Goodfellow and Williams, 1983).

Reports focusing on biological control often provide partial or full protection. Thus far, many studies recommend using IDM as an applied disease control strategy combining chemical and biological antagonists on crops (AbuQamar et al., 2017) along with breeding and biotechnology programs (AbuQamar et al., 2016; Sham et al., 2017). We propose that isolates of *S. globosus* such as the one we studied is an excellent candidate as BCA for the management of this devastating disease.

Future ‘omics’ analyses will advance our understanding of the biology of *T. punctulata*, and will shed light on the complex *T. punctulata*-date palm interaction. Ultimately, our long-term goal is to develop future strategies to effectively manage the black scorch disease using environmentally sustainable strategies. Although many actinomycete isolates have been identified from a wide range of environmental habitats to manage other fungal pathogens in other countries (Goodfellow and Williams, 1983; Doumbou et al., 2001; El-Tarabily and Sivasithamparam, 2006), this is the first report of the antagonistic activity of an actinomycete strain against any *Thielaviopsis* species, isolated from UAE soils, or elsewhere.

## Author Contributions

ES, RI, KE-T, and SAQ designed the research. KE-T and SAQ supervised the study. ES, AS, ZS, YA, and KE-T performed *in vitro* and *in vivo* experiments. ES, AS, KE-T, and SAQ performed *in vivo* greenhouse experiments. AS and SAQ developed the phylogenetic analysis. RI, KE-T, and SAQ analyzed the data. ZS and YA assisted with experiments and/or data evaluation. K-ET and SAQ wrote the manuscript. All authors critically revised the manuscript and approved the final version.

## Conflict of Interest Statement

The authors declare that the research was conducted in the absence of any commercial or financial relationships that could be construed as a potential conflict of interest.
