# Supplementary material for: Streptomyces globosus UAE1, a Potential Effective Biocontrol Agent for Black Scorch Disease in Date Palm Plantations
Source: Front Microbiol. 2017 Jul 31;8:1455. doi: 10.3389/fmicb.2017.01455 (PMC5534479; doi:10.3389/fmicb.2017.01455)
Supplement: Supplementary file 1 [file Data_Sheet_1.PDF]

Supplementary Figures

Streptomyces globosus UAE1, a Potential Effective Biocontrol Agent for Black Scorch Disease in Date Palm Plantations

Esam Eldin Saeed, Arjun Sham; Zeinab Salmin; Yasmeen Abdelmowla; Rabah Iratni; Khaled El-Tarabily*, and Synan AbuQamar*

*** Correspondence:**
Dr. Khaled El-Tarabily: ktarabily@uaeu.ac.ae
Dr. Synan AbuQamar: sabuqamar@uaeu.ac.ae

Supplementary Figures

Figure S1. Inhibition of *T. punctulata* mycelial growth by the BCA. (A) Cut-plug method, and (B) dialysis membrane overlay technique. In (B) the diffusible antifungal metabolites-producing *S. globosus* (isolate #7) compared to the non-diffusible antifungal metabolites-producing *Streptomyces* sp. (isolate #25).

Figure S2. Production of volatile antifungal metabolites and chitinase enzyme by the BCA. (A) Effect of *T. punctulata* mycelial growth by non-volatile antifungal metabolites producing *S. globosus* (isolate #7; middle panel) compared to the volatile antifungal metabolites producing *Streptomyces* sp. (isolate #25; lower panel). FMEA plates (right), are either colonized by no *Streptomyces* sp. (C; upper panel), *S. globosus* (isolate #7; middle panel) or *Streptomyces* sp. (isolate #25; lower panel), respectively and (B) production of chitinase enzyme by the chitinase-producing *Micromonospora* sp. (isolate# 4) compared to chitinase-non-producing *S. globosus* (isolate #7).

Figure S3. Inhibitory growth effect of Cidely® Top fungicide on *T. punctulata*. Growth inhibitory effect on *T. punctulata* using Cidely® Top (in ppm). Data were collected 15 d after inoculation.

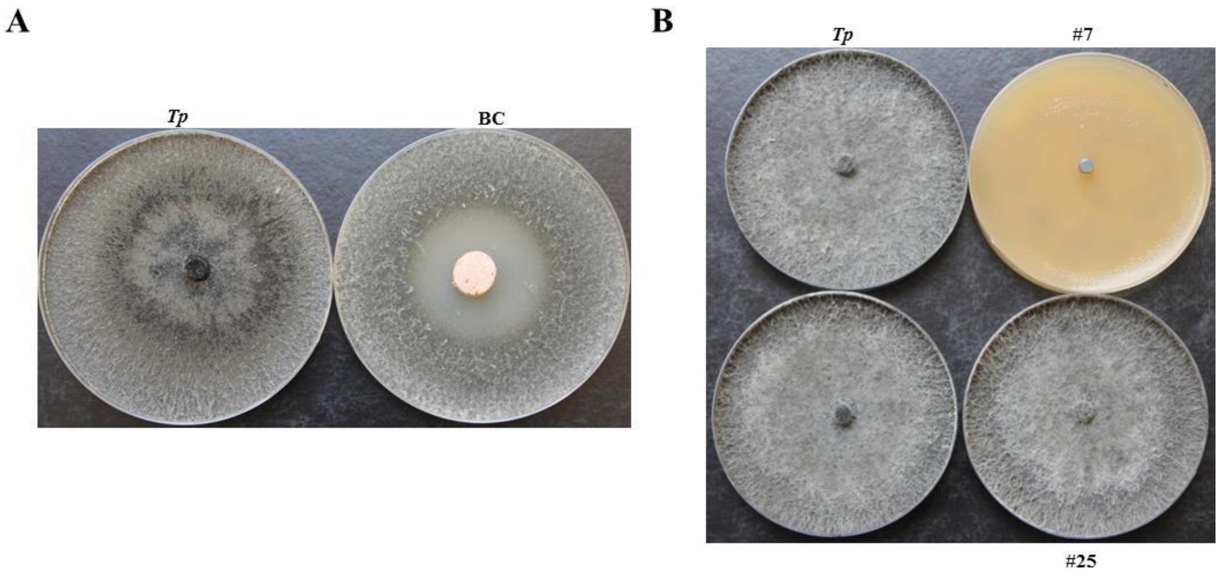

Figure S1. Inhibition of *T. punctulata* mycelial growth by the BCA. (A) Cut-plug method, and **(B)** dialysis membrane overlay technique. In **(B)** the diffusible antifungal metabolites-producing *S. globosus* (isolate #7) compared to the non-diffusible antifungal metabolites-producing *Streptomyces* sp. (isolate #25).

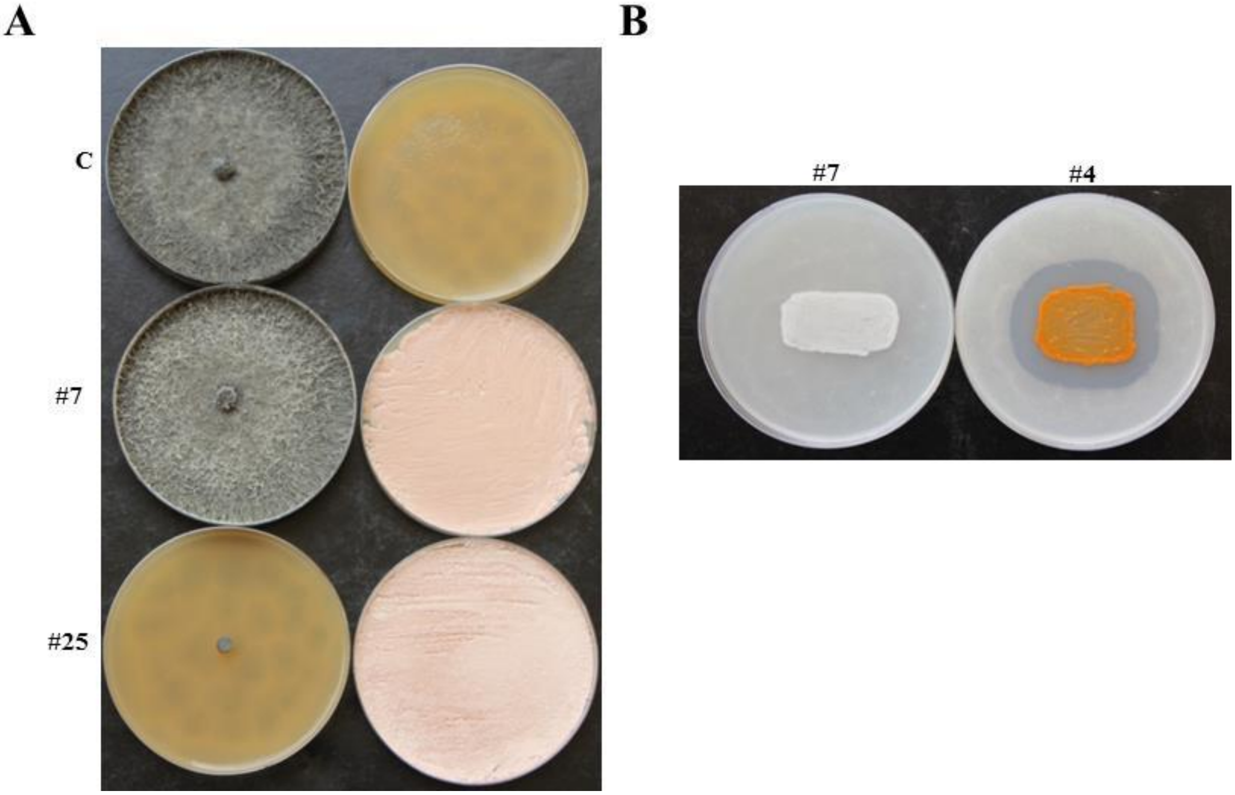

Figure S2. Production of volatile antifungal metabolites and chitinase enzyme by the BCA. (A) Effect of *T. punctulata* mycelial growth by non-volatile antifungal metabolites producing *S. globosus* (isolate #7; middle panel) compared to the volatile antifungal metabolites producing *Streptomyces* sp. (isolate #25; lower panel). FMEA plates (right), are either colonized by no *Streptomyces* sp. (C; upper panel), *S. globosus* (isolate #7; middle panel) or *Streptomyces* sp. (isolate #25; lower panel), respectively and **(B)** production of chitinase enzyme by the chitinase-producing *Micromonospora* sp. (isolate# 4) compared to chitinase-non-producing *S. globosus* (isolate #7).

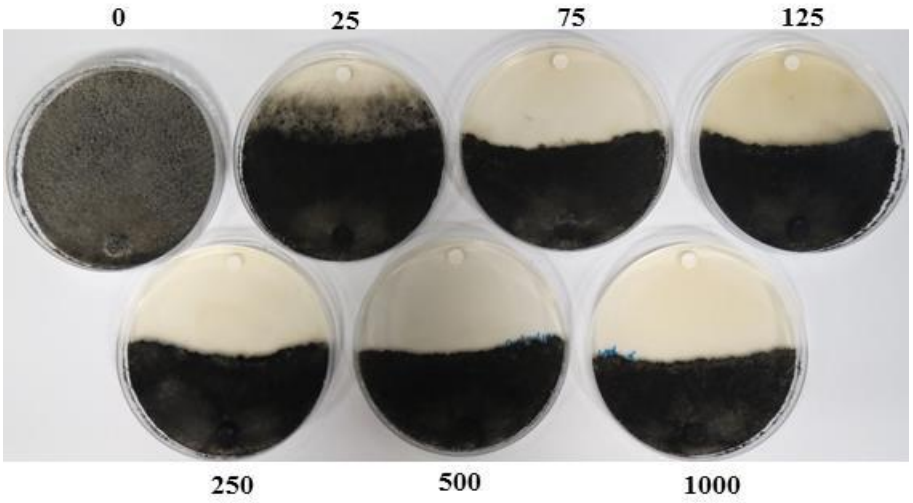

Figure S3. Inhibitory growth effect of Cidely® Top fungicide on *T. punctulata*. Growth inhibitory effect on *T. punctulata* using Cidely® Top (in ppm). Data were collected 15 d after inoculation.
